# Evaluation of tibial tunnel placement in single case posterior cruciate ligament reconstruction: reducing the graft peak stress may increase posterior tibial translation

**DOI:** 10.1186/s12891-019-2862-z

**Published:** 2019-11-07

**Authors:** Zhiqiang Wang, Yan Xiong, Qi Li, Gang Chen, Zhong Zhang, Xin Tang, Jian Li

**Affiliations:** 1 Department of Orthopaedic Surgery, West China Hospital, Sichuan University, No. 37, Guoxue Alley, Chengdu, 610041 People’s Republic of China; 2 Department of Joint Surgery, Suining Central Hospital, No. 127, West Desheng Rd., Chuanshan District, Sichuan, 629000 Sichuan Province People’s Republic of China

**Keywords:** Killer turn, Posterior cruciate ligament, Reconstruction, Finite element, Biomechanics

## Abstract

**Background:**

The killer turn has been documented as the primary drawback of posterior cruciate ligament (PCL) reconstruction. Fanelli advocated placing the tibial tunnel outlet in the inferior lateral part of the PCL fovea to reduce the killer turn. This study aimed to confirm the validity of Fanelli’s viewpoint regarding PCL reconstruction technique and to assess the specific Fanelli tunnel area on the inferior lateral part of the PCL fovea.

**Methods:**

The geometrical data of the model were obtained by nuclear magnetic resonance (MRI) and computerized tomography (CT), with images taken from a healthy Chinese volunteer. The three-dimensional finite element model of the knee joint was established using Mimics, Geomagic Studio, 3-matic, and Ansys software. The finite analysis was performed after the material behavior, contact and boundary conditions, and loading were defined. The drawer tests were simulated with a posterior tibial load of 134 N at 0°, 30°, 60°, and 90° knee flexion. The PCL peak stress and tibial translation were recorded and compared among the 30 distinct tibial tunnel loci over a range of angles from 0° to 90°.

**Results:**

In the area (Fanelli area, 5–20 mm inferior and 5–10 mm lateral to the PCL anatomical insertion), the lowest PCL peak stress in all sites with different flexion angles was lower than that of the PCL anatomical insertion site. The lowest PCL peak stress with different knee flexion angles was observed in the following location: 10 mm inferior and 5 mm lateral to the PCL anatomical insertion. In the Fanelli area, the tibial translations of three sites were lower and those of other sites were higher than that of the PCL anatomical insertion site.

**Conclusions:**

PCL reconstruction in the Fanelli area, especially 10 mm inferior and 5 mm lateral to the PCL anatomical insertion, could reduce the peak stress of the graft and may reduce the killer turn. However, whether the posterior stability of the knee is affected needs to be further studied.

## Background

Posterior cruciate ligament (PCL) transtibial reconstruction is a commonly surgery in sports medicine. Although double bundle PCL reconstructions can be equivalent to the outcomes for ACL reconstructions [1], the results of single bundle PCL reconstruction have been less satisfactory compared to anterior cruciate ligament reconstructions [2–5]. A systematic review of PCL reconstructions revealed that the overall failure rate of PCL single-bundle reconstruction was 12.5%. Only 50 to 82% of the patients who underwent PCL reconstruction were able to return to preinjury activity level [6]. Hence, there are several questions regarding the causes of PCL reconstruction failure. The “killer turn” has been documented as a primary drawback for bone-patellartendon-bone (BTB) PCL reconstruction [7–12].

Over the last 15 years, Fanelli introduced a new viewpoint, that is, placing the tibial tunnel outlet in the inferior lateral part of the PCL fovea (Fanelli tunnel) to reduce the killer turn [13]. Good clinical and functional outcomes have been reported after long-termfollow-up [14–16]. However, so far, we have not found any biomechanical evidence for the Fanelli tunnel yet. Additionally, the specific location coordinates (distance and direction from the anatomical stop) of the tunnel were unclear.

The present study aimed (1) to confirm the validity of Fanelli’s viewpoint regarding PCL reconstruction technique and (2) to determine the specific tibial tunnel placement on the inferior lateral part of the PCL fovea through three-dimensional (3D) finite element (FE) analysis. We hypothesized that Fanelli’s viewpoint regarding PCL reconstruction technique is correct- placing the tibial tunnel outlet in the inferior lateral part of the PCL fovea could reduce the graft peak stress.

## Methods

### Establishment of three-dimensional finite element model of the knee joint

A volunteer (175 cm, 75 kg) provided informed consent for inclusion in this study. The geometrical data of the models developed herein were obtained using Siemens ingenuity core 64-slice spiral computed tomography (CT) and 1.5-T dual gradient nuclear magnetic resonance imaging (MRI, Siemens MAGNETOM Aera). CT images were used for the 3D reconstruction of bone structures (tibia, fibula, femur, patella). MR images were used for the 3D reconstruction of soft structures (ligaments, menisci, tendons, and cartilage). The knee was immobilized in full extension inside a plaster cast, avoiding any movement during MRI and CT scanner image acquisition. The scanning range was upper and lower 15 cm of the knee joint. The images were taken in sagittal, coronal, and transverse planes. The layer thicknesses of CT and MRI scanning were 1 mm and 0.6 mm, respectively. The images were saved in DICOM format.

CT and MRI scanning data in DICOM format were imported into Mimics 21 (Materialise Inc., Leuven, Belgium), a medical image processing software. The 3D models of the bone tissue (tibia, fibula, femur, patella) and soft structures (ligaments, menisci, tendons, and cartilage) were obtained. Using the image segmentation and automatic extraction of the system, accurate 3D segmentation and model reconstruction were performed. The established 3D models of the bone, articular cartilage, ligament, and menisci were saved in STL format.

The 3D models of the ligament, articular cartilage, and meniscus in STL format were converted to CT scanning data space. The position of the cartilage, ligament, and meniscus in the skeleton 3D model was adjusted to conform to the anatomical structure of the knee joint and to integrate the 3D FE model of the knee joint. Registration alignment of the 3D model of the soft tissue surface was performed using SolidWorks 2018 software (Dassault Systemes Inc., France) based on two different modal data.

**Fig. 1:**
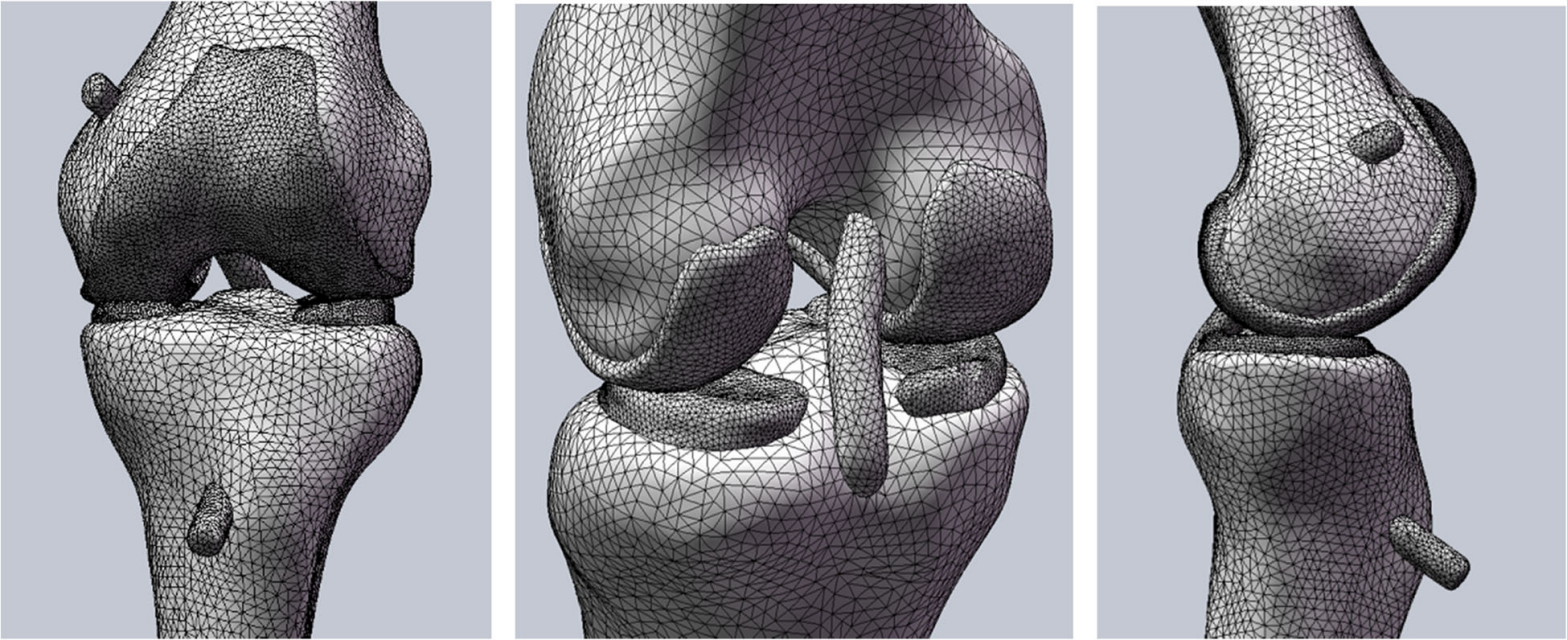
Abaqus finite element analysis software is used to mesh the three-dimensional geometric model of the knee joint. The model was divided into 30,111 units and 36,012 nodes

Surface mesh editing tool using Geomagic 2013 software (Geomagic Inc., USA) was used to make the model smoother and suppler, allowing the model to achieve high-quality surface. Abaqus FE analysis software was used to mesh the 3D geometric model of the knee joint. The model was divided into 30,111 units and 36,012 nodes (Fig. 1).

### The establishment of different flexion angle models

After PCL simulated reconstruction, the femur was fixed, and the tibia was rotated backward to 30°, 60°, and 90° using a software, respectively. The spatial position of the ligaments of the knee joint was adjusted to achieve a better spatial anastomosis. The stress concentrated and the potential interference zone for late calculation were corrected. Different flexion angle models at 30°, 60°, and 90° were established.

### Material properties and boundary conditions

Since stiffness of the bone is much higher than that of the relevant soft tissues, bones were assumed to be rigid. According to the previous reports [17, 18], we set the material properties of the articular cartilage and meniscus as homogeneous, continuous, and isotropic elastic material with elastic moduli of 5 MPa and 59 MPa and Poisson ratios of 0.46 and 0.49 for the articular cartilage and meniscus, respectively [17, 18]. Lastly, we set the ligament as homogeneous, continuous, and isotropic elastic material with an elastic modulus of 215.3 MPa and a Poisson ratio of 0.4.

To make the model closer to the entity, the two ends of the main ligament and its anatomical attachment points were set as joint contact connection; the surface of the articular cartilage was fixed with the surface of the bone tissue; and the anterior and posterior corners of the meniscus and the outer edge of the medial meniscus were fixed with the edge of the tibial plateau to simulate the attachment of the meniscus to the tibial plateau. The contact property between the cartilage and meniscus was considered as nonlinear friction-free contact [17, 18].

### Tibial and femoral tunnel placement

**Fig. 2:**
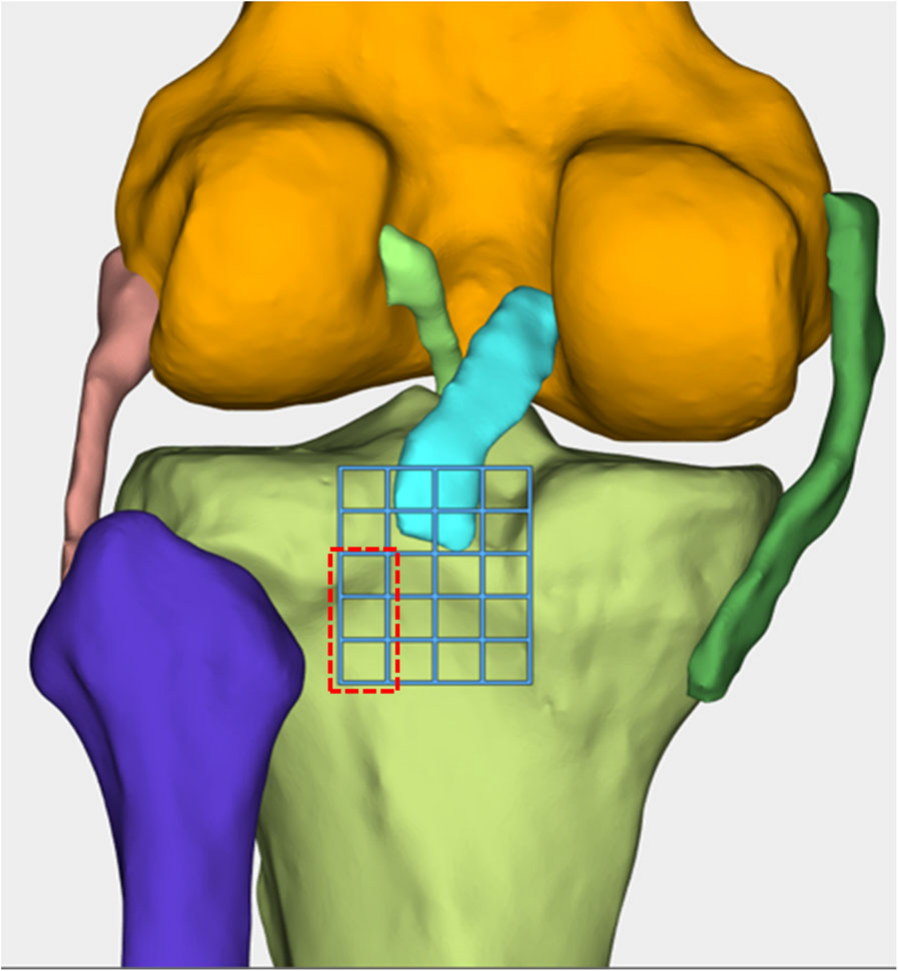
On the proximal tibia, 5-mm squares were drawn in the proximal-distal direction and the medial-lateral direction, with the posterior cruciate ligament’s (PCL) anterolateral bundle’s anatomic footprint at the center. The femoral tunnel was located at the femoral footprint of the anterolateral bundle of the PCL. A total of 30 distinct tibial tunnel placement loci were obtained (29 novel loci plus anatomical center). Fanelli area (the red dotted line box) located 5–20 mm inferior and 5–10 mm lateral to the posterior cruciate ligament anatomical insertion site

**Fig. 3:**
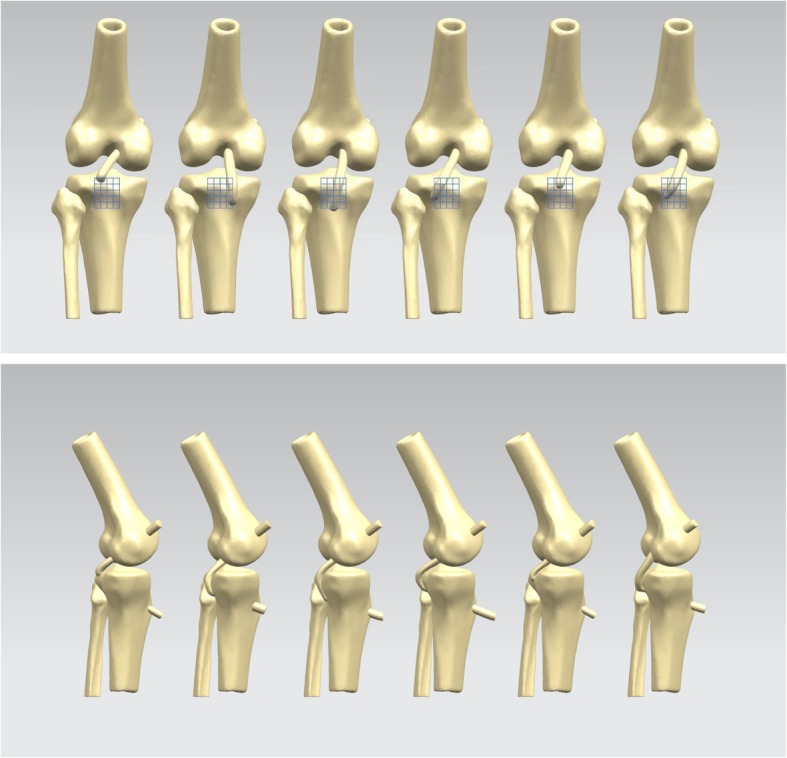
Morphology of the posterior cruciate ligament (PCL) reconstruction with different tibial tunnels in the proximal-distal direction and the medial-lateral direction relative to the anatomic footprint. Only PCL in boundary position of the proximal-distal direction and the medial-lateral direction and the anatomical footprint were shown

On the proximal tibia, 5-mm squares were drawn in the proximal-distal direction and the medial-lateral direction, with the PCL anterolateral bundle’s anatomical footprint at the center (Fig. 2). The femoral tunnel was located at the femoral footprint of the anterolateral bundle of the PCL. A total of 30 distinct tibial tunnel placement loci (29 novel loci plus anatomical center) and 12 tibial tunnel placement loci, located in the inferior lateral part of the anatomical footprint, were observed (Fanelli area). The femoral tunnel and tibial tunnel were assumed to be circular with a diameter of 9 mm. For each location of the PCL graft tibial tunnel placement, a distinct FE simulation was developed, representing the baseline configuration of the tibial graft positioned in the anatomical footprint, in addition to a separate simulation for each of the 30 divergent tunnel loci (Fig. 3).

### Simulated knee joint kinetics

**Fig. 4:**
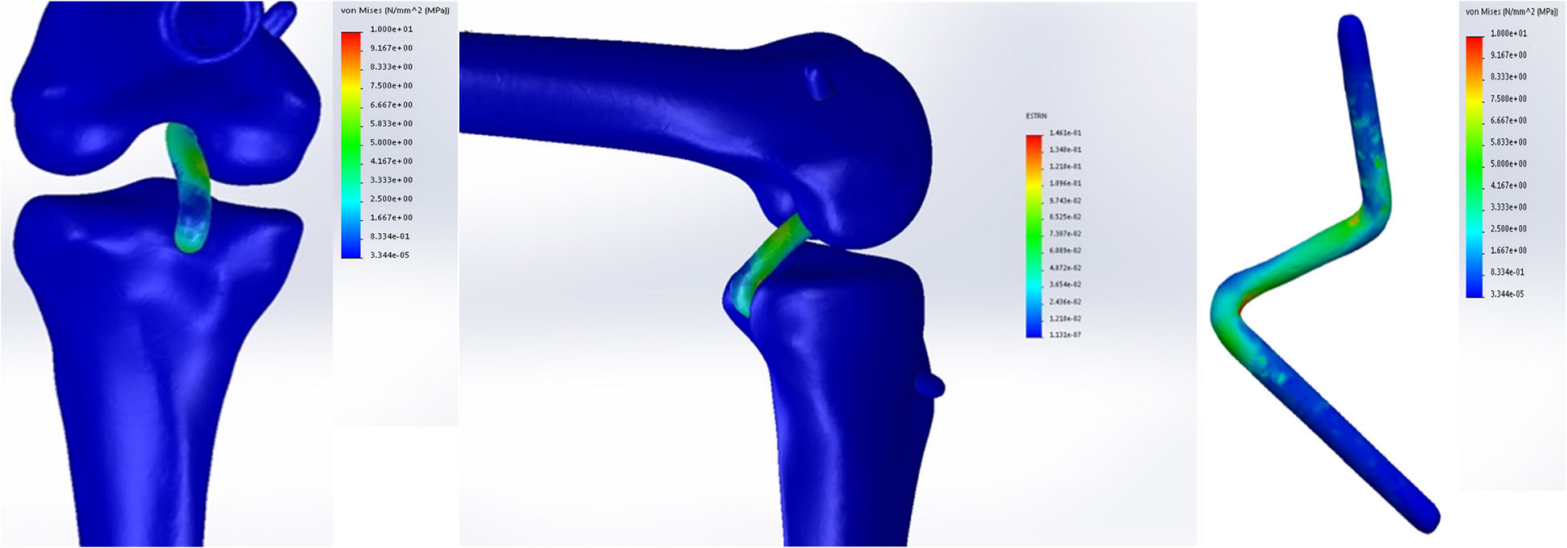
In the genuflex model with 90° flexion, a posterior tibial load of 134 N was applied to the proximal tibia at different knee flexion. The peak stresses of the PCL graft were recorded and compared among the 30 distinct tibial tunnel loci

**Fig. 5:**
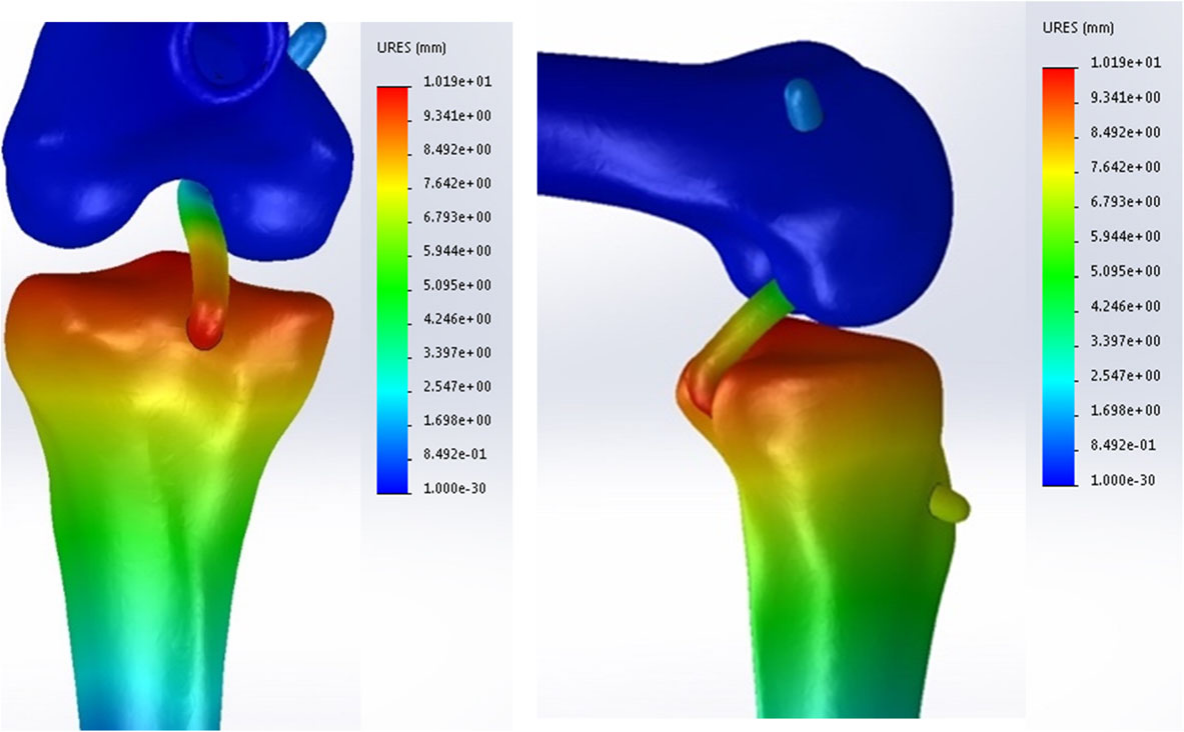
In the genuflex model with 90° flexion, a posterior tibial load of 134 N was applied to the proximal tibia at different knee flexion. The tibial translations were recorded and compared among the 30 distinct tibial tunnel loci

The drawer tests were used to assess knee joint laxity and global knee biomechanics in each model, representing a different tibial graft placement. A posterior tibial load of 134 N [19–22] was applied to the proximal tibia at 0°, 30°, 60°, and 90° knee flexion, respectively. Additional details related to the kinetics and kinematics of the drawer test simulation were described elsewhere [23]. The peak stress of the PCL graft (Fig. 4) and the knee joint stability (Fig. 5) were recorded and compared among the 30 distinct tibial tunnel loci over a range of angles from 0° to 90°.

## Results

**Fig. 6:**
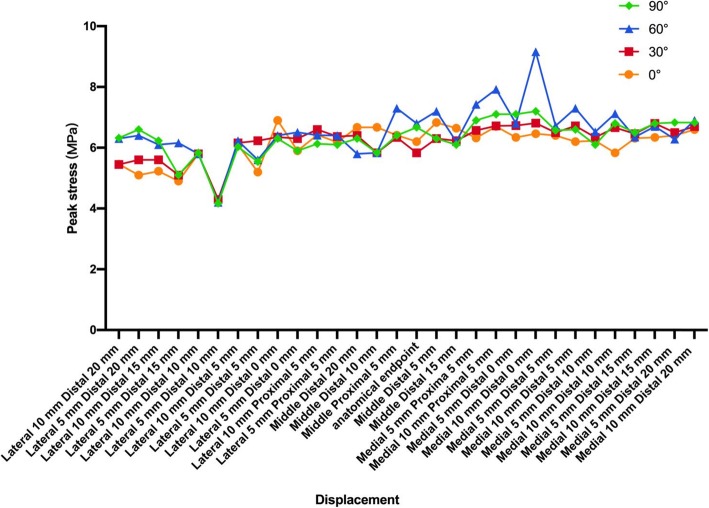
Line chart of the PCL peak stress of all sites with different flexion angles

The peak stress of the tunnel graft created at the lateral aspect of the PCL anatomical insertion sites was lower than that of the medial aspect. In the area (5–20 mm inferior and 5–10 mm lateral to the PCL anatomical insertion site, Fig. 2), the lowest PCL peak stress of all sites with different flexion angles was lower than that of the PCL anatomical insertion site (Fanelli area). This indicates that the graft is not loaded here, further noting that this is not an anatomic position. The lowest PCL peak stress range with different knee flexion angles was 4.2–4.3 MPa. All the sites appeared in the following location: 10 mm inferior and 5 mm lateral to the PCL anatomical insertion site, which was 3.94–13.99% lower than the peak stress of the PCL anatomical insertion site (Fig. 6).

**Fig. 7:**
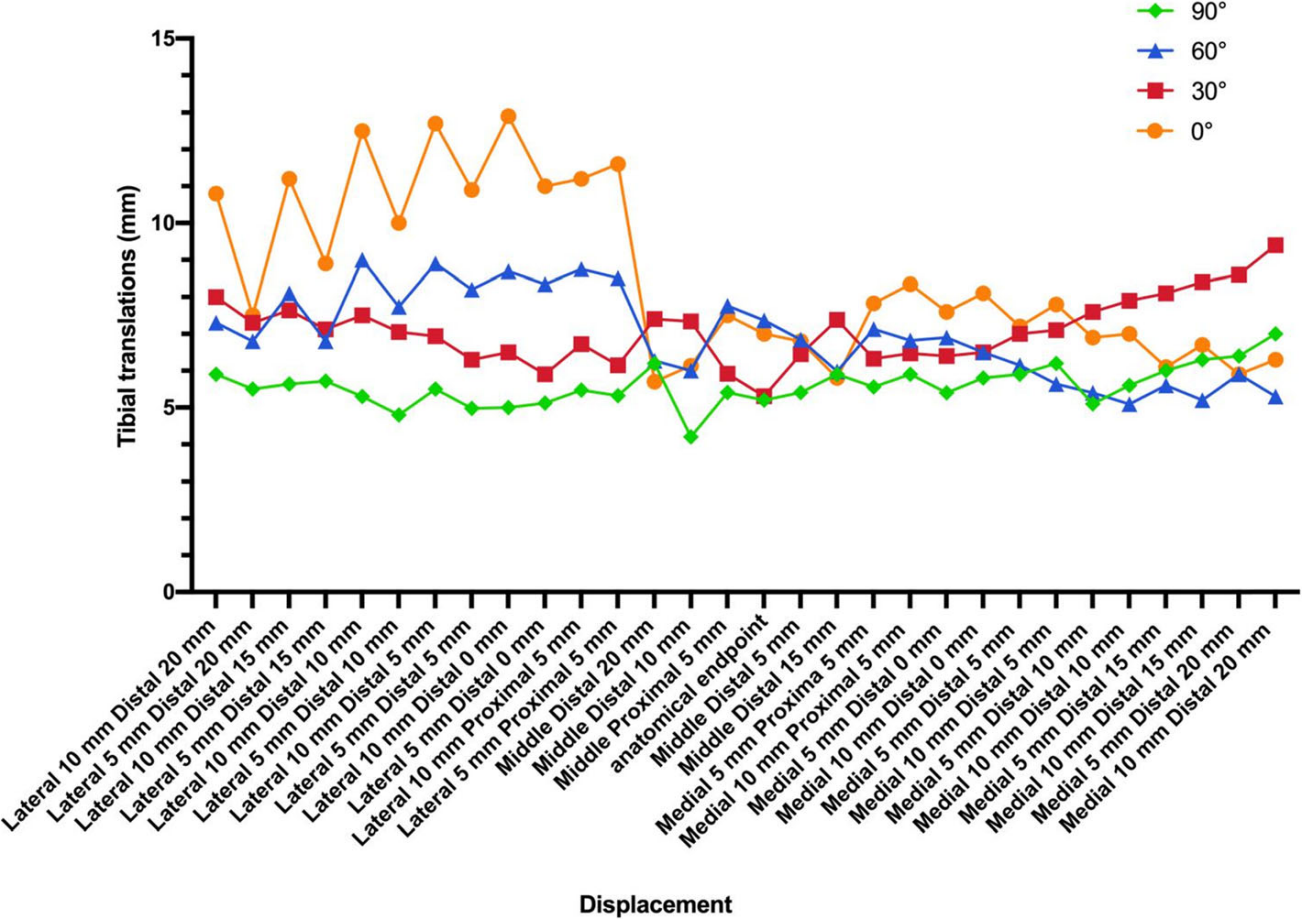
Line chart of the tibial translation of all sites with different flexion angles

The tibial translation of the PCL anatomical insertion site was 5.2–7.4 mm with different knee flexion angles. For tibial translations, only three sites in the Fanelli area were lower than that of the PCL anatomical insertion site. The tibial translations of the other sites inside the Fanelli area and outside the Fanelli area were higher than that of the PCL anatomical insertion site (Fig. 7).

## Discussion

The present study performed an finite element analysis on the knee’s biomechanical influence on the PCL grafts reconstructed using different tibial tunnels. We found that the tibial tunnel in the 5–20 mm inferior and 5–10 mm lateral to the PCL anatomical insertion site location could reduce the PCL peak stress with different flexion angles. The lowest PCL peak stress tunnel placement had the following location: 10 mm inferior and 5 mm lateral to the PCL anatomical insertion site. However, the tibial tunnels in the Fanelli area may slight increase their tibial translation compared to the PCL anatomical insertion site. This result confirms Fanelli’s viewpoint. PCL reconstruction in the Fanelli area could reduce the peak stress of the graft and may reduce the killer turn.

For transtibial PCL reconstruction, the graft will form an acute angle around the proximal posterior tibia, which results in excess forces and graft abrasion at the proximal margin of the tibial tunnel exit for BTB grafts [7–12]. According to Fanelli’s viewpoint, placing the tibial guide in the inferior lateral aspect of the PCL anatomical insertion site could convert the acute angle between the graft and tunnel to two smooth obtuse angles on the posterior aspect of the tibia [13] and could reduce the local peak stress and graft abrasion. The specific tibial tunnel placement (10 mm inferior and 5 mm lateral to the PCL anatomical insertion site) is the extension of the posteromedial bundle of PCL. It coincides with the medial groove and lateral cartilage [24]. Creating a tibial tunnel in this placement, the PCL graft could pass the PCL’s tibial footprint to the medial femoral condyle. It may reduce the friction between the graft and the bone, reduce PCL peak stress, and prevent graft sliding.

In the literature, there are few biomechanical studies on the comparison between the PCL non-anatomical endpoints and anatomical endpoints. Markolf et al. [25] conducted a biomechanical study to measure graft forces and knee laxity at five knee flexion angles with a tibial tunnel located at the center of the PCL’s tibial footprint, 5 mm medial and lateral to the central location. The results showed that with the exception of slightly higher graft forces recorded with the medial tunnel beyond 65° passive knee flexion, drilling the tibial tunnel 5 mm medial or lateral to the center of the PCL’s tibial footprint had no significant effects on the biomechanical characteristics of the reconstructed knee. LaPrade [26] concluded that reaming of the tibial tunnel proximally had a high risk of injuring the posterior meniscal roots. Galloway et al. [27] reconstructed the PCL on cadaver specimens and compared the biomechanical results of five different tibial attachments, the PCL tibial insertion and 5-mm offset in the medial, lateral, proximal, and distal directions. The results showed that changes in the tibial attachment had minor effect on knee stability. Significant differences were found at 30^o^ and 60^o^ between the medial and lateral tibial attachments. This result is basically consistent with our results. However, there was no further comparative study on the other areas such as the lateral/ inferior, medial/inferior, lateral/ superior, and medial/superior areas except the 5 points.

FE analysis is an important means of biomechanical analysis, which can solve the problem of insufficient cadaver specimens in traditional biomechanical experiments. Compared with traditional cadaveric experimentation, a wide selection of points (30 tunnels) is possible in the 3D FE test. Another distinct advantage of the present computational formulation is the ability to assess small incremental differences in graft placement [21].

Our study has the following limitations. First, this is a FE analysis with only a single subject’s anatomic geometry; hence, cadaver biomechanical test will be needed in performing the next step. Second, in the present study, only tibial tunnels were compared; thus, the effects of PCL femoral insertion were not included. Third, we found that the tibial tunnels in the Fanelli area may slightly increase their tibial translation compared to that of the PCL anatomical insertion site; hence, whether it will affect the posterior stability of knee needs to be further validated by clinical research.

## Conclusions

In conclusion, PCL reconstruction in the Fanelli area, especially 10 mm inferior and 5 mm lateral to the PCL anatomical insertion site, could reduce the peak stress of the graft and may reduce the killer turn. However, posterior stability to the knee was increased and this requires further study.

## Data Availability

The datasets used and/or analyzed during the current study are available from the corresponding author on reasonable request.

## Abbreviations

3D: finite element
CT: computed tomography
FE: three-dimensional
MRI: magnetic resonance imaging
PCL: posterior cruciate ligament
